# Efficient Photosynthesis of Value-Added Chemicals by Electrocarboxylation of Bromobenzene with CO_2_ Using a Solar Energy Conversion Device

**DOI:** 10.3390/ijms251910608

**Published:** 2024-10-01

**Authors:** Yingtian Zhang, Cui Gao, Huaiyan Ren, Peipei Luo, Qi Wan, Huawei Zhou, Baoli Chen, Xianxi Zhang

**Affiliations:** Shandong Provincial Key Laboratory/Collaborative Innovation Center of Chemical Energy Storage, School of Chemistry and Chemical Engineering, Liaocheng University, Liaocheng 252059, China; lcuytz@126.com (Y.Z.); gaocuigc@163.com (C.G.); rhy17862548687@163.com (H.R.); luopeipeigood@163.com (P.L.); w15689397767@163.com (Q.W.); xxzhang3@126.com (X.Z.)

**Keywords:** CO_2_ carboxylation, electrosynthesis, electrocarboxylation, bromobenzene, photovoltaic, dye-sensitized solar cells

## Abstract

Solar-driven CO_2_ conversion into high-value-added chemicals, powered by photovoltaics, is a promising technology for alleviating the global energy crisis and achieving carbon neutrality. However, most of these endeavors focus on CO_2_ electroreduction to small-molecule fuels such as CO and ethanol. In this paper, inspired by the photosynthesis of green plants and artificial photosynthesis for the electroreduction of CO_2_ into value-added fuel, CO_2_ artificial photosynthesis for the electrocarboxylation of bromobenzene (BB) with CO_2_ to generate the value-added carboxylation product methyl benzoate (MB) is demonstrated. Using two series-connected dye-sensitized photovoltaics and high-performance catalyst Ag electrodes, our artificial photosynthesis system achieves a 61.1% Faraday efficiency (FE) for carboxylation product MB and stability of the whole artificial photosynthesis for up to 4 h. In addition, this work provides a promising approach for the artificial photosynthesis of CO_2_ electrocarboxylation into high-value chemicals using renewable energy sources.

## 1. Introduction

The rapidly growing consumption of fossil fuels is bringing significant environmental crises, such as the “greenhouse effect”, to the world today [1,2]. The release of CO_2_, the most important greenhouse gas, is increasing at an alarming rate of 2 ppm per year with a total concentration exceeding 420 ppm currently [3]. This has a significant impact on the climate and normal life, and urgently needs to be addressed. In addition, from the view of abundance, non-toxicity, renewability, and economy, CO_2_ is also an ideal and useful C1 building block in synthetic chemistry [4]. Efficiently converting CO_2_ into value-added chemicals can reduce CO_2_ emissions and effectively alleviate the pressure of fossil energy demand, making it a strategic research topic for scientists [5]. Electrochemical technology which deals with a clean reagent, the electron, can convert CO_2_ into value-added chemicals under mild and safe conditions. Hence, considerable attention has been devoted to the electroreduction of CO_2_ to generate value-added fuels [6] such as CO [7,8], CH_4_ [9], and ethanol [10,11] and the electrocarboxylation of CO_2_ with organic molecules such as organic halides [12,13,14,15], alkenes [16,17], and ketones [18,19,20] to generate high-value-added chemicals (such as aromatic carboxylic acids, aromatic carboxylic esters, and their derivatives).

In addition, the energy crisis is also becoming increasingly severe; world energy demand continues to increase and will expand by 48% between 2012 and 2040 [21]. The conversion efficiency of solar energy into chemical energy in natural photosynthesis is generally not high, and most plants do not exceed 1%. Therefore, compared to natural photosynthesis, the energy conversion efficiency of artificial photosynthesis systems needs to be higher than 1%. At present, the energy conversion efficiency of most artificial photosynthesis is higher than that of natural photosynthesis (about 10%, with a few exceeding 20%) [22,23]. Hence, this research direction is promising. Using renewable energy sources such as naturally available solar energy to convert CO_2_ into high-value-added fuels and chemicals is an elegant solution to address environmental and energy crises, providing an effective strategy for closing the carbon cycle and meeting future energy demands [24]. Thus, this approach, called “artificial photosynthesis”, is well-reported for the sunlight-driven electroreduction of CO_2,_ generating fuels such as CO [25], CH_4_ [26], and methanol [27]. For instance, Schreier et al. reported electroreduction of CO_2_ into CO using series-connected perovskite photovoltaics solar cells [25] and triple-junction GaInP/GaInAs/Ge solar cells [28] with simulated sunlight to provide the voltage and current needed for electroreduction of CO_2_. Compared to the encouraging achievements in the field of artificial photosynthesis for CO_2_ electroreduction into fuels, research on artificial photosynthesis for CO_2_ electrocarboxylation with organic molecules to value-added chemicals such as aromatic carboxylic acids, aromatic carboxylic acid esters, and their derivatives is very scarce, making it highly needed [29,30]. Moreover, the electrocarboxylation of organic halides with CO_2_ is one of the important reactions to gain carboxylic acids or carboxylic acid ester [13,14,15,31,32,33,34,35].

**Figure 1:**
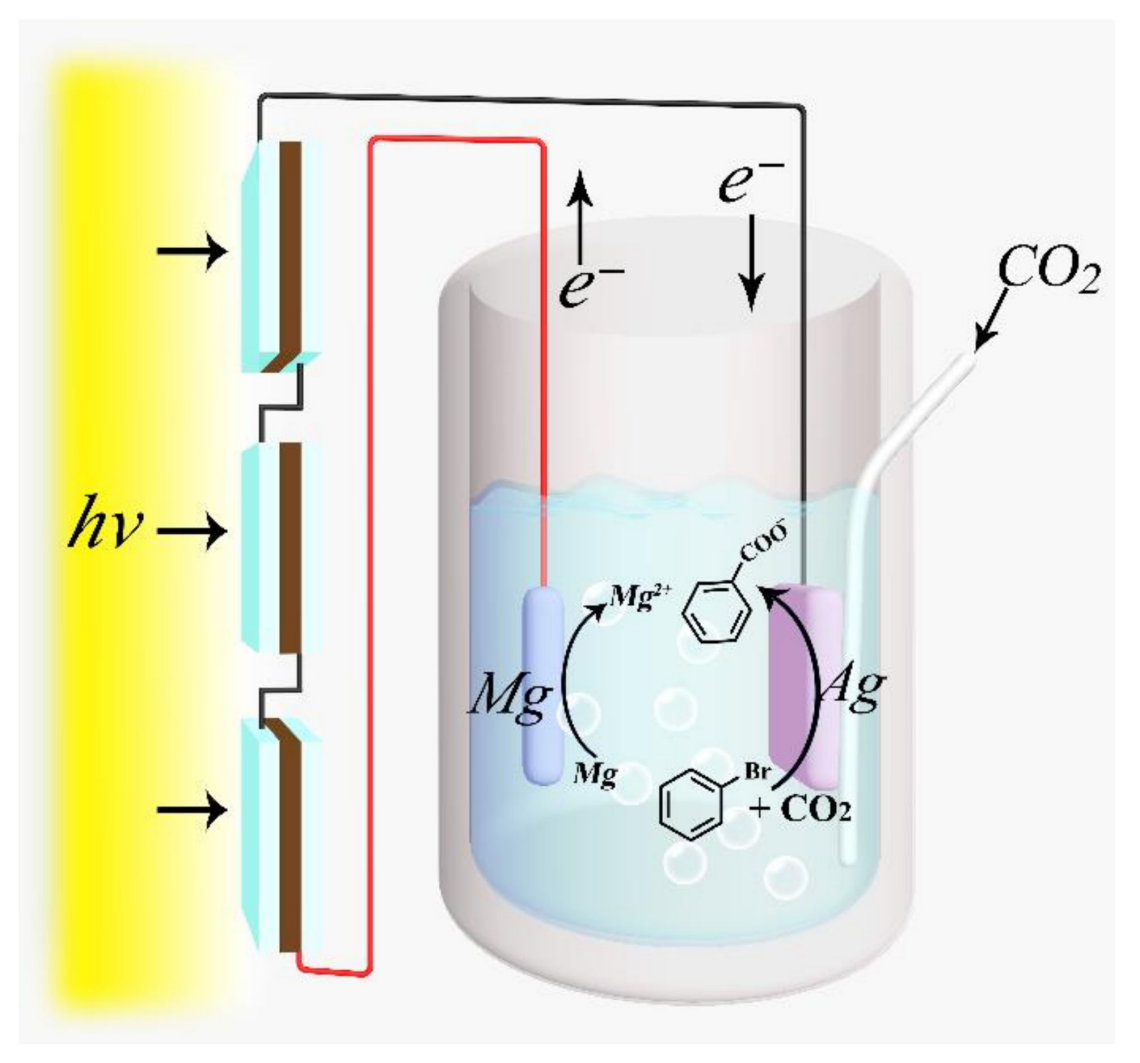
The diagrammatic sketch of artificial photosynthesis for electrocarboxylation of bromobenzene (BB) with CO_2_ to a carboxylation product.

Furthermore, dye-sensitized solar cells (DSCs) are clean, green, and pollution-free solar cells, and solar photovoltaic technology is a clean way to generate electric power directly from solar radiation [36]. Here, inspired by the natural photosynthesis of green plants (where separate steps of light reaction and (dark) carbon fixation) and the artificial photosynthesis for CO_2_ electroreduction to fuel, we extend our previous work in DSCs [37] and electrocarboxylation of organic molecules with CO_2_ [14,15,38,39]. In this study, we demonstrate the effective artificial photosynthesis for electrocarboxylation of bromobenzene (BB) with CO_2,_ driven solely by simulated sunlight, to obtain the corresponding carboxylation product methyl benzoate (MB), which is a commonly used spice for preparing Yilan and Wanxiangyu fragrances and is also widely used for food essences, such as strawberries. Using series-connected dye-sensitized photovoltaics and excellent performance silver catalyst electrode, we achieve a 61.1% Faraday efficiency (FE) for MB (a detailed diagram of the artificial photosynthesis is shown in Figure 1). Our study offers a very promising approach for producing high-value chemicals from the artificial photosynthesis of BB with CO_2_ using renewable energy sources.

## 2. Results and Discussion

**Scheme 1:**
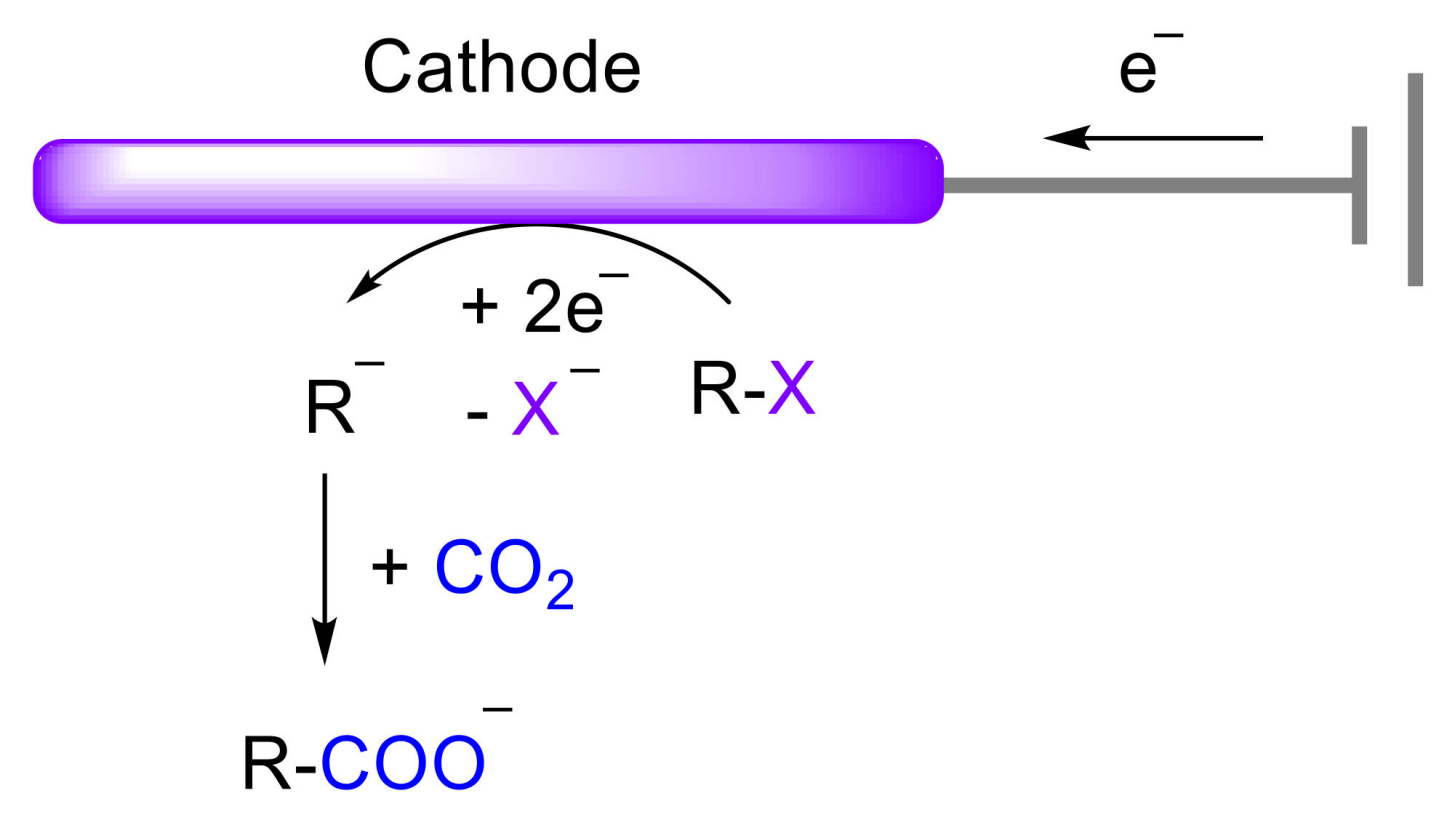
A schematic diagram of the general mechanism for the electrocarboxylation of organic halides with CO_2_.

As mentioned above, the electrocarboxylation of organic halides with CO_2_ is one of the most important reactions for producing carboxylic acids or carboxylic acid esters [13,14,15,31,32,33,34,35]. Meanwhile, BB is the simplest halogenated aromatic compound; therefore, the electrocarboxylation of BB with CO_2_ is chosen for the CO_2_ artificial photosynthesis herein. Generally, when the reduction potential of the organic halides is more positive than that of CO_2_, the organic halides are first reduced at the cathode. Thus, the mechanism for electrocarboxylation of aryl halides (RX) with CO_2_ generally includes a two-electron cleavage of the C–X bond in RX to generate carbanion intermediates (R^−^), and then the nucleophilic reaction with CO_2_ (seen in Scheme 1) [31,32,33,40,41]. In addition, studies show that the Ag cathode displays significant electrocatalytic activity in the electrocarboxylation of organic halides with CO_2_ [32,33,41]. Therefore, the Ag electrode was selected as a working electrode for the electrocarboxylation of BB with CO_2_.

### 2.1. Cyclic Voltammograms of BB

**Figure 2:**
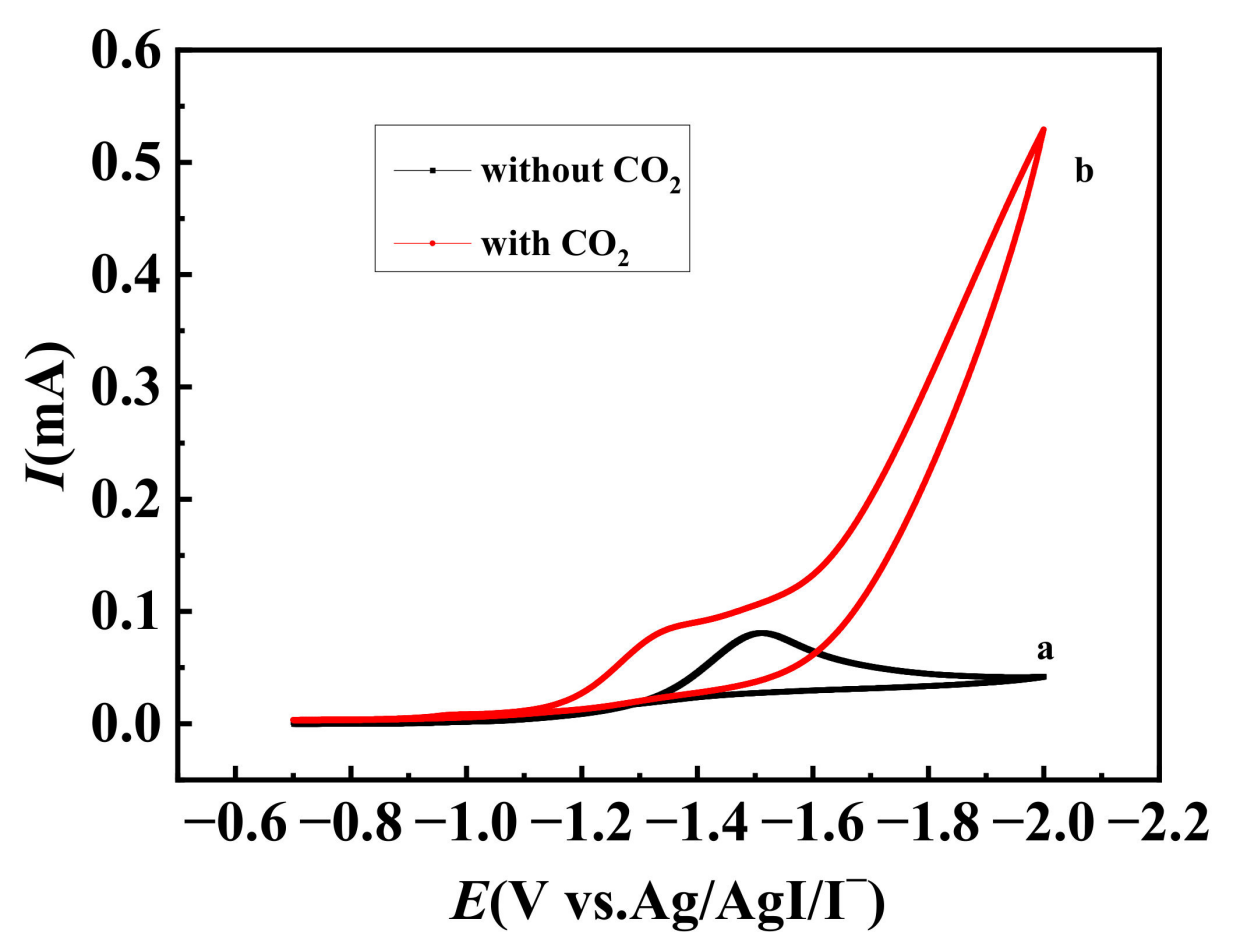
Cyclic voltammograms of 5 mM BB were obtained on a Ag electrode with a scan rate of 0.1 V/s in 0.1 M TEABF_4_–DMF in the absence of CO_2_ (a) and the presence of CO_2_ (b).

Cyclic voltammograms were first conducted in a three-electrode system to study the electrocarboxylation reaction and are shown in Figure 2. The working electrode was the Ag electrode (d = 2 mm), the auxiliary electrode was Pt, and the reference electrode was Ag/AgI/0.1 mol/L tetrabutylammonium iodide (TBAI) in DMF. As shown in curve (a) of Figure 2, the electroreduction of BB reveals a single irreversible reduction peak at −1.51 V (vs Ag/AgI/I^−^) at the Ag electrode in the presence of N_2_, corresponding to the two-electron reductive cleavage of the carbon–halogen bond of BB to carbanion intermediates [32,33]. The current starts to increase from −1.00 V (vs Ag/AgI/I^−^). Different behavior was observed after the solution was saturated with CO_2_, as shown in curve (b) of Figure 2. The reduction peak potential shifts significantly more positively to −1.34 V (vs Ag/AgI/I^−^), and the current starts to increase from −0.90 V (vs Ag/AgI/I^−^) (Figure 2). The above results indicate the existence of a rapid chemical reaction between the electroreduced intermediate and CO_2_ [31,32,33,40], consistent with the general mechanism for the electrocarboxylation of organic halides with CO_2_ as shown in Scheme 1.

### 2.2. Electrocarboxylation of BB with CO_2_ by Constant Potential Electrolysis

**Scheme 2:**
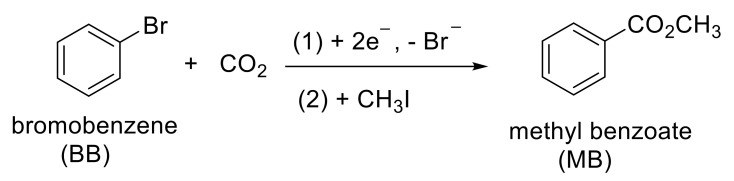
Schematic diagram of the conversion of CO_2_ by electrocarboxylation of BB with CO_2_ to obtain a carboxylic acid ester.

**Figure 3:**
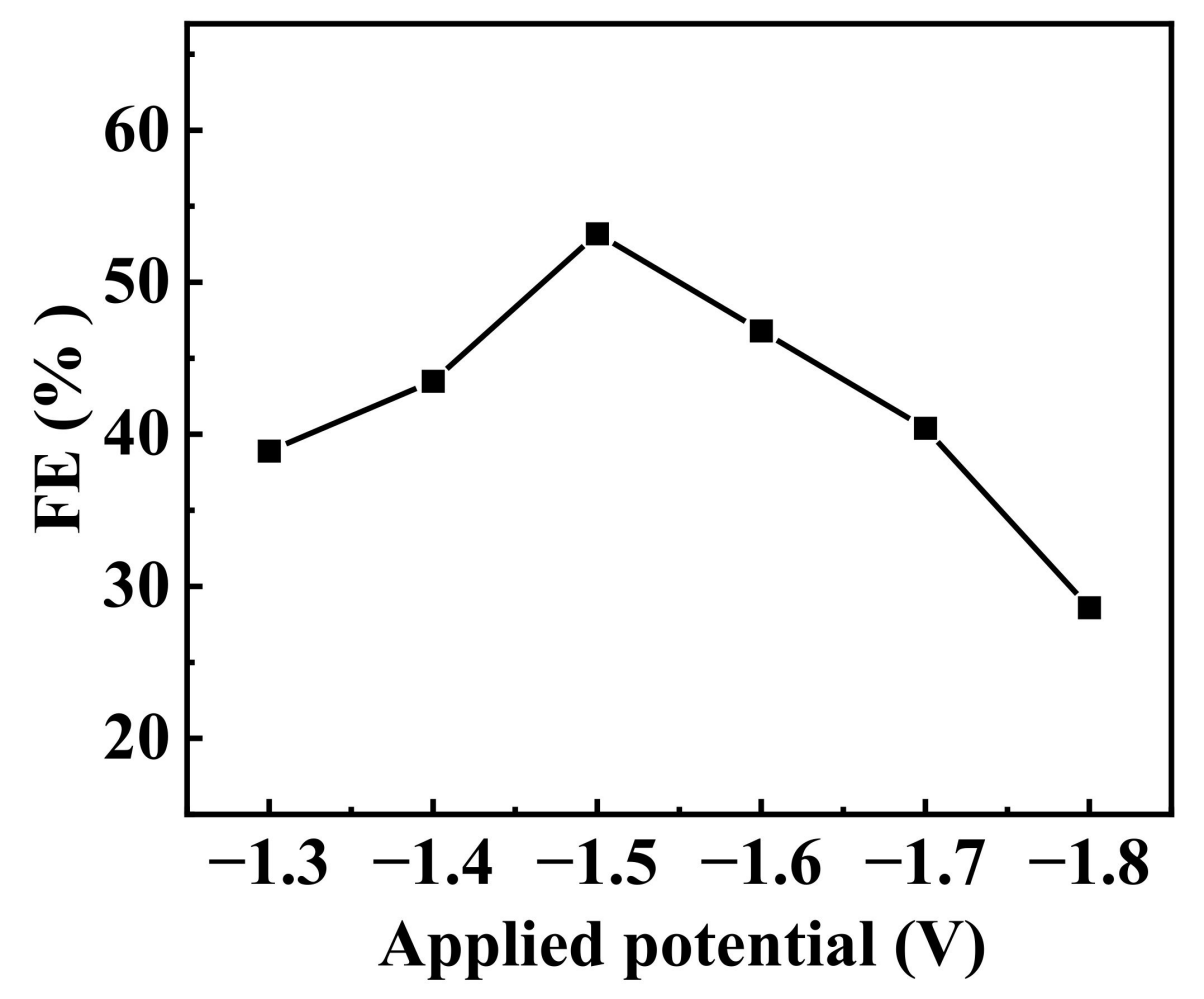
The Faraday efficiency of electrocarboxylation of BB with CO_2_ under different applied potentials.

To further investigate the electrocarboxylation of BB with CO_2_ and obtain the electrocarboxylation product, we studied dark reactions by constant potential electrolysis using a potentiostat (CHI760E (Shanghai Chenhua Instruments Company, Shanghai, China), an instrument that can accurately control the working electrode potential in the electrolysis) with a three-electrode system [32,42]. In these experiments, electrolysis was carried out in CO_2_-saturated DMF containing 0.1 M BB and 0.1 M tetrabutylammonium iodide (TBAI) supporting electrolyte in an undivided cell with a Ag cathode, Mg anode, and Ag/AgI/I^−^ reference electrode until 2.0 F/mol of charge (*Q*) past the electrolysis cell at 0 °C under atmospheric pressure. In addition, to detect the carboxylation products using gas chromatography–mass spectra (GC-MS) and gas chromatography (GC), the electrolyzed liquids were esterified with CH_3_I and converted into corresponding carboxylate ester. GC-MS analysis showed that the carboxylation product methyl benzoate (MB) was obtained (the electrocarboxylation schematic diagram is seen in Scheme 2). The amount of the carboxylation product MB was formulated by a standard curve using GC with the internal standard method, as shown in Figure S1. The FE was calculated using the following equation:

(1) $$FE\%=\frac{n_{MB}\times z\times F}{Q}$$

where *n* is the actual number of moles obtained for the carboxylation product MB; *F* is the Faraday constant; *z* equals 2 for MB, which is the number of electrons transferred during the electrocarboxylation of BB to MB; and *Q* is the passed charge of electricity, which here is 193 C. The dark reaction was tested under different potentials, and the results are shown in Figure 3. The FE values under −1.3, −1.4, −1.5, −1.6, −1.7, and −1.8 V were 38.9%, 43.5%, 53.2%, 46.8%, 40.4%, and 28.6%, respectively. The results indicate that the FE of MB showed a trend of first increasing and then decreasing with the negative shift of electrolysis potential, and the highest FE was 53.2% at −1.5 V. This is because when the applied potential moves more negatively, on the one hand, the proportion of Faraday current in the system increases, thereby improving the yield of the target carboxylation product MB. On the other hand, excessive negative shifts of applied potential may also lead to the occurrence of unwanted side reactions, thereby affecting the generation of product MB. Therefore, there exists an optimal electrolysis potential that maximizes the yield of MB, which here is −1.5 V, corresponding to the reduction peak potential of BB under N_2_ conditions (curve (a) of Figure 2).

### 2.3. CO_2_ Artificial Photosynthesis for CO_2_ Electrocarboxylation with BB

**Figure 4:**
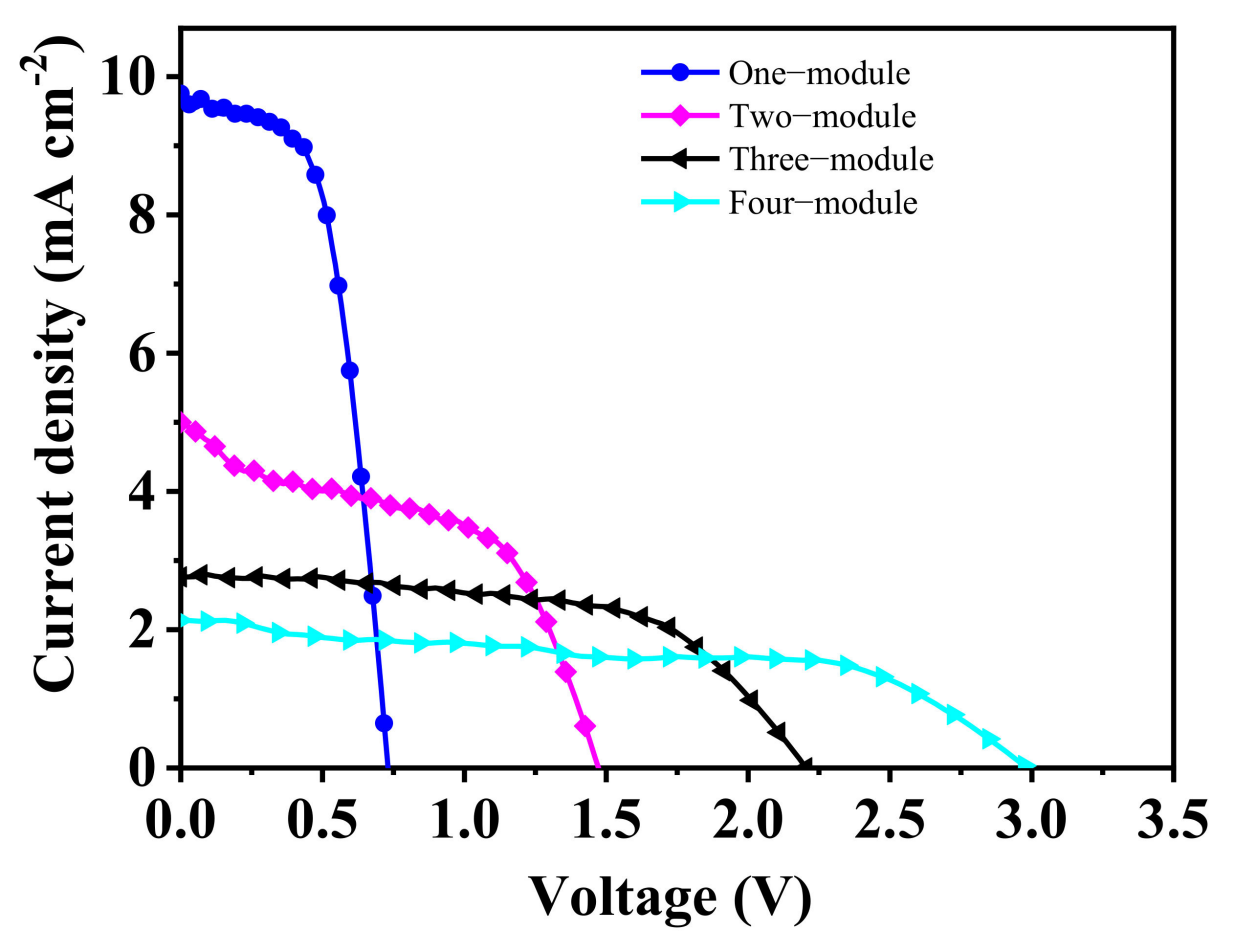
The J–V curve of photovoltaic modules under simulated sunlight (AM1.5, 100 mW cm^−2^).

Moreover, DSC is a photochemical cell, which is low-cost, green, and colorful. It can convert solar energy into electricity and convert CO_2_ into useful chemicals. This technology not only reduces the CO_2_ levels in the environment but also the whole process is green and pollution-free. In the CO_2_ artificial photosynthesis experiments, firstly, DSCs were prepared according to our previous study [37], and a stacking–separating strategy was adopted to assemble the series-connected DSCs module. The composition of the DSCs is as follows: an FTO acts as a conductive substrate, a N719-sensitized TiO_2_ mesoporous film (12 μm) acts as a photoanode, I_3_^−^/I^−^ acts as the electrolyte, and pyrolytic Pt/FTO acts as the counter electrode. The DSCs were encapsulated by a polymer film. Aluminum foil is used as an electron collector between every DSC. Then, we assembled DSCs into photovoltaic modules to provide different potentials for CO_2_ conversion. We studied one, two, three, and four modules as the power source. The performance of the photovoltaic module under simulated sunlight (AM1.5, 100 mW cm^−2^) is shown in Figure 4. Measuring the J–V curve (Figure 4) usually involves applying the resistance loads of different resistance values to a digital source meter to measure the voltage across the ends of the photovoltaic module and the current passing through it. When the resistance load is particularly small, the current in the circuit is relatively large and changes smoothly with the increase in resistance load, corresponding to the flat region of the J–V curve. When the load increases to a certain extent, the current tends to decrease linearly, that is, in a way that is equivalent to the drastically decreasing region. When the load is particularly large, the current tends to zero, and the voltage corresponds to the open–circuit voltage. As seen from the J–V curve (Figure 4), we know that the photovoltaic parameters of the one–module are as follows: the short–circuit current density (J_sc_) is 9.67 mA·cm^−2^, the open−circuit voltage (V_oc_) is 0.73 V, the fill factor (FF) is 0.59, and the power conversion efficiency (PCE) is 4.18%. The photovoltaic parameters of the two–module version are 5.00 mA·cm^−2^ for the J_sc_, 1.47 V for the V_oc_, 0.49 for the FF, and 3.64% for the PCE. The photovoltaic parameters of the three–module version are 2.83 mA·cm^−2^ for the J_sc_, 2.20 V for the V_oc_, 0.57 for the FF, and 3.56% for the PCE. The photovoltaic parameters of the four–module version are 2.17 mA·cm^−2^ for the J_sc_, 2.98 V for the V_oc_, 0.55 for the FF, and 3.55% for the PCE.

Next, we conducted the CO_2_ artificial photosynthesis for BB with CO_2_ by assembling DSCs in series to provide different potentials. This process (photochemical cell + CO_2_ electrocarboxylation conversion), using sunlight to generate value-added chemicals, can be called artificial photosynthesis. The part of DSCs can be called the photoreaction. The part of the CO_2_ electrocarboxylation conversion reaction can be called the dark reaction. The photoreaction is linked to the dark reactions, as shown in Figure 1. The dark reaction was carried out in an undivided cell containing 10 mL DMF, 0.1 M BB, and 0.1 M TBAI supporting electrolyte, using Ag as the cathode and Mg as the anode until 2.0 F/mol of charge (*Q*) passed through the electrolysis cell at 0 °C under atmospheric pressure. The CO_2_ gas was bubbled through the solution (near the Ag electrode). The entire diagrammatic sketch of the artificial photosynthesis is shown in Figure 1. The general process of artificial photosynthesis includes the following steps. The sunlight was irradiated onto the photoanode (FTO/dye-sensitized meso-TiO_2_ film). The ground state electrons of the dye (N719) were excited to the excited state, as shown in Equation (2). The excited state electrons were injected into the conductive band of TiO_2_ and passed through FTO to the external circuit. The external circuit electrons flowed to the Ag cathode. Considering that the reduction potential of BB is more positive than that of CO_2_ on the Ag electrode [32,33], the BB would first undergo a two-electron reduction to generate the corresponding dehalogenated carbanion; afterward, it could easily react with CO_2_ around the Ag catalyst to produce a dehalogenated carboxylation product (Equation (3)). If BB and CO_2_ were continuously added to the dark reaction system, the reaction would continue. For the Mg anode, it would undergo an oxidation reaction to lose two electrons and generate Mg^2+^ (Equation (4)). The external circuit electrons passed through FTO to the cathode of DSCs. The presence of I_3_^−^ around the Pt catalyst would result in a two-electron reduction (Equation (5)). The oxidized dye could be reduced by I^−^, as in Equation (6).

(2) $$Dye+hv\to Dye^{+}+e^{-}$$

(3) $$C_{6}H_{5}Br+CO_{2}+2e^{-}\to C_{6}H_{5}COO^{-}+Br^{-}$$

(4) $$Mg\to Mg^{2+}+2e^{-}$$

(5) $$I_{3}^{-}+2e^{-}\to 3I^{-}$$

(6) $$2Dye^{+}+3I^{-}\to 2Dye+I_{3}^{-}$$

**Figure 5:**
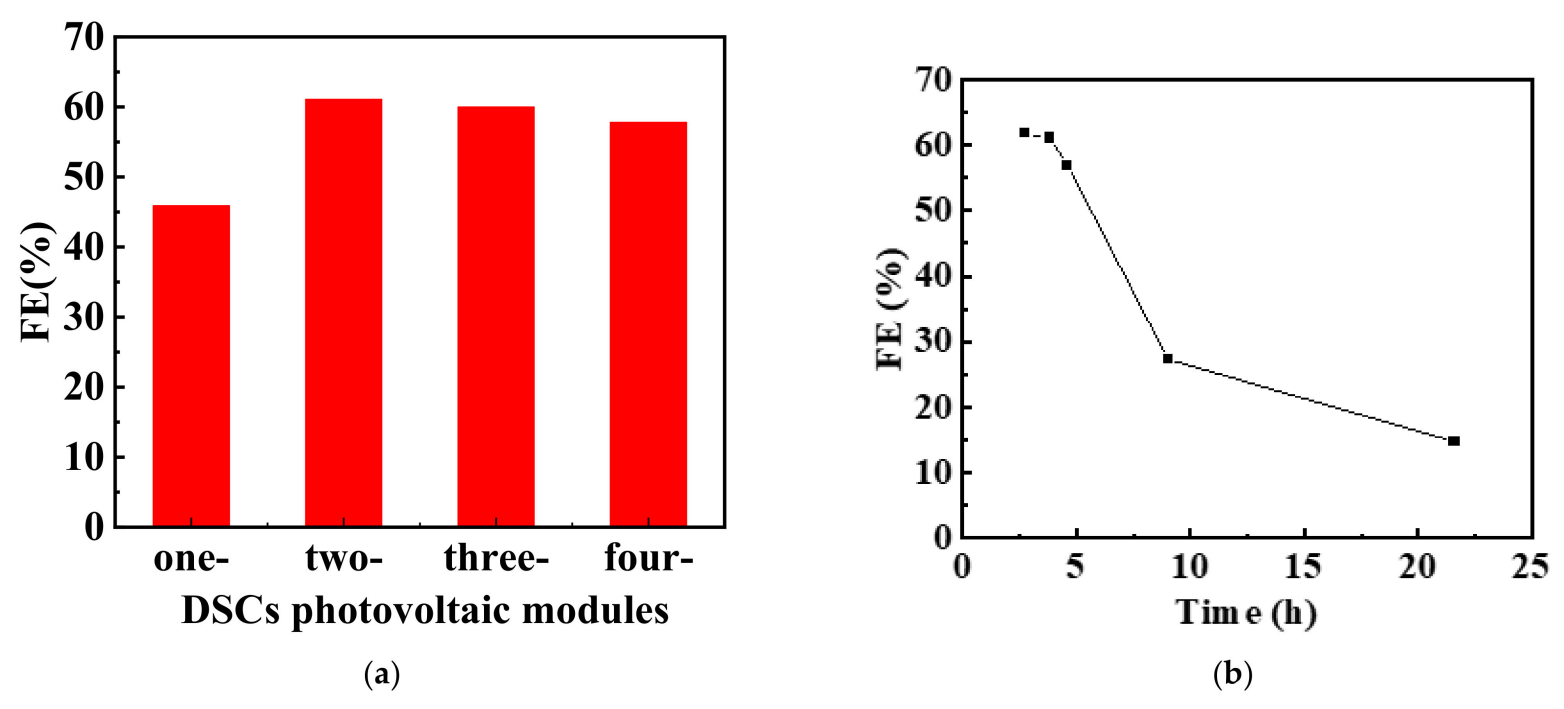
The CO_2_ artificial photosynthesis for CO_2_ electrocarboxylation and the stability. (**a**) The Faraday efficiency of CO_2_ artificial photosynthesis for CO_2_ electrocarboxylation with BB using a photovoltaic module under simulated sunlight (AM1.5, 100 mW cm^−2^). (**b**) The stability of FE in CO_2_ artificial photosynthesis for CO_2_ electrocarboxylation with BB.

To further investigate the efficiency of the whole artificial photosynthesis, the charge (*Q*) passed the artificial photosynthesis was collected through the following method. The multimeter (UNT-T, UT61E) was connected to a computer using an RS232 serial port (Ugreen Group Limited, Shenzhen, China). The collected current value of the reaction was transmitted to the computer every 0.1 s. The data were imported into Microsoft Office Excel 2016 to calculate the real-time quantity of charge. In addition, to detect the carboxylation products using GC-MS and GC, the electrolyzed liquids were esterified with CH_3_I and converted into the corresponding carboxylate ester. After artificial photosynthesis and esterification, GC-MS analysis showed that the carboxylation product MB (the molecular architecture seen in Scheme 2) was obtained. The amount of the carboxylation product MB was formulated by the standard curve using GC with the internal standard method, as shown in Figure S1. The FEs of artificial photosynthesis based on one-, two-, three-, and four-module versions were also calculated using Equation (1), and the results are shown in Figure 5a. The FEs of the system based on one-, two-, three-, and four-module versions were 45.9%, 61.1%, 60.0, and 57.8%, respectively, and the highest FE was obtained based on two-module DSCs. Moreover, the FE (61.1%) based on two-module DSCs in artificial photosynthesis was higher than that (53.2%) applied 1.5 V (vs Ag/AgI/I^−^) for ordinary electrocarboxylation reaction, as shown in Figure 3.

The stability of the whole artificial photosynthesis was then tested with two series-connected photovoltaic modules, and the result is shown in Figure 5b. The FE is still close to the initial value after 3.8 h, which indicates that the artificial photosynthesis system we designed is effective. However, as the reaction time continues to increase, the FF value shows a downward trend; therefore, improvement is needed for long-term stability. To further improve the stability duration of the system, the high stability of Ag catalytic electrodes needs to be further addressed. The process of chemical equilibrium also requires a design to ensure the continuous circulation and stability of the reaction. In addition, there is a risk of liquid leakage during the long-term operation of liquid DSCs, and the development of solid-state DSCs is also a solution to maintain long-term stability.

## 3. Materials and Methods

### 3.1. Materials

*N*,*N*-dimethylformamide (DMF) was dried over 4 Å molecular sieves, and other reagents were used as received. Bromobenzene (BB, 99% purity) and Methyl benzoate (MB, 99% purity) were purchased from Beijing J andK Technology Co., Ltd. (Beijing, China. CH_3_I was bought from Zhengzhou Alpha Chemical Co., Ltd. (Zhengzhou, China). Tetraethylammonium tetrafluoroborate (TEABF_4_, 99% purity) was bought from Alfa aesar Chemical Co., Ltd. (Shanghai, China). The other reagents were gained from Sinopharm Chemical Reagent. Co., Ltd. (Beijing, China). Gas chromatography–mass spectra (GC-MS) analyses were recorded on an Agilent 7000D instrument (Agilent Technologies Co., Ltd., Santa Clara, CA, USA). The product FE was obtained through Shimadzu GC-2014C gas chromatography (GC, Shimadzu Manufacturing Co., Ltd., Kyoto, Japan). The power conversion efficiency curves (J–V) of the DSCs were obtained by a solar energy testing system (IV5, PV Measurements, Inc., Washington, DC, USA) equipped with a solar simulator (CEL-S500-T5, Beijing China Education Au-light Co., Ltd., Beijing, China) to provide simulated sunlight. Constant potential electrolysis and cyclic voltammograms were carried out with a CHI760E electrochemical workstation (Shanghai Chenhua Instruments Company, Shanghai, China). Photoelectrochemical electrolysis was performed using a DSCs module with a solar simulator (CEL-S500-T5, Beijing China Education Au-light Co., Ltd., Shanghai, China) to provide simulated sunlight and we used a multimeter (Ulide Technology (China) Co., Ltd., Dongguan City, China) connected to a Lenovo computer to obtain the charge (*Q*) past the circuit.

### 3.2. General Procedure for the Cyclic Voltammograms

The electroanalytical experiments were conducted using three-electrode systems in an undivided glass cell. The Ag catalytic (d = 2 mm) electrode, platinum wire (which has good conductivity and chemical stability to ensure the accuracy and stability of experiments), and Ag/AgI/0.1 mol/L tetrabutylammonium iodide (TBAI) in DMF were used as the working electrode, auxiliary electrode, and reference electrode, respectively. The experimental medium was 10 mL of DMF solvent containing 0.1 mol/L of TEABF_4_ supporting electrolyte to improve the conductivity of the solution. All the experiments were conducted under normal temperatures and pressures.

### 3.3. General Procedure for Electrocarboxylation of BB with CO_2_ by Constant Potential Electrolysis

In a typical experiment, constant potential electrolysis was performed in a mixture solution of BB (0.1 mol/L) and TBAI (0.1 mol/L) in DMF solvent (10 mL) at 0 °C with continuous CO_2_ bubbling in an undivided cell equipped with a Ag (8 cm^2^) cathode, a magnesium (Mg) rod sacrificial anode, and a Ag/AgI/0.1 mol/L TBAl reference electrode at a certain potential until 2.0 F/mol of charge (*Q*) had passed through the electrolysis cell at 0 °C under atmospheric pressure. Once the electrolysis was accomplished, the generalized products were esterified by adding CH_3_I (0.3 mL) and anhydrous K_2_CO_3_ (0.3 g) at 55 °C for 5 h. Subsequently, the solution was acidified with aqueous HCl (0.8 mol/L) and then extracted by diethyl ether. The organic layers were combined and then dried over anhydrous MgSO_4_. Then, the target product methyl benzoate (MB) was obtained after filtration and rotary evaporation. Finally, the identification of the target product MB was performed using GC–MS and comparing GC retention time for the target carboxylation product with that of a commercially available MB with a purity of 99%. The FE of the target product MB was obtained through GC with the calibration curves (see Supplementary Figure S1 online). In the GC detection, the internal standard method was employed to obtain the quantification of MB, and decane was used as the internal standard.

### 3.4. Preparation and Assembly of DSCs

The Pt catalytic electrode, which serves as the counter electrode, is prepared by pyrolysis. In a typical experiment, first, spray the H_2_PtCl_6_ aqueous solution (8% wt) onto the cleaned FTO conductive glass (substrate). Heat H_2_PtCl_6_ on FTO on a hot plate at 80 °C to evaporate the solvent; afterward, heat to 450 °C to pyrolyze to produce Pt on FTO, and then the counter electrode is ready. Second, print the TiO_2_ layer (10 μm) on FTO glass using TiO_2_ paste, and then heat the film at 500 °C for 60 min to remove the binder. According to reference [37], the photoanode is treated with a TiCl_4_ aqueous solution (0.04 mol/L) for 30 min to produce amorphous TiO_2_. Next, heat the TiO_2_ film again at 500 °C for 30 min to form a coating layer, thereby increasing the surface area. When the film is cooled to 80 °C (which is conducive to the full infiltration and adsorption of the dye), immerse it in N719 dye in an alcohol solvent (10^−5^ mol/L) for 20 h to prepare a dye-sensitized photoanode. Third, apply a clean, shape-specific Surlyn hot melt film onto the dye-sensitized photoanode, and next place the Pt electrode onto the Surlyn hot melt film. A stacking–separating strategy is adopted to assemble the series-connected DSCs module. So, afterward, stack the sandwich device of photoanode/Surlyn film/Pt electrode together using clamps. Heat the sandwich device in a drying oven at 180 °C for 3 min to melt Surlyn film; then, remove it and cool it to room temperature to complete the encapsulation of DSCs. Fourth, a 0.5 mL electrolyte is dropped into the small holes in the FTO glass to fill the inner of the DSCs. Then, package the dye-sensitized photoanode/electrolyte/Pt electrode sandwich device by sealing the small hole with UV glue and irradiating it with a UV lamp for 10–20 min. Finally, DSCs are connected in series (while the power conversion efficiency curve (J–V) was measured) for later use.

### 3.5. General Procedure for CO_2_ Artificial Photosynthesis for CO_2_ Electrocarboxylation with BB

In a typical experiment, CO_2_ artificial photosynthesis was performed using DSCs as the power instead of CHI760E, and the DSCs connected in series were linked to carboxylation reactions, according to Figure 1. The DSCs connected in series were illuminated under simulated sunlight (AM1.5, 100 mW cm^−2^). The carboxylation reactions were carried out in a mixture of BB (0.1 mol/L) and TBAI (0.1 mol/L) in DMF solvent (10 mL) at 0 °C with continuous CO_2_ bubbling in an undivided cell equipped with a two-electrode system (a Ag (8 cm^2^) cathode and a Mg rod sacrificial anode) at a certain photovoltaic module until 2.0 F/mol of charge (*Q*) past the electrolysis cell under atmospheric pressure. The multimeter (UNT-T, UT61E), connected to the computer, was used to collect the current value of the reaction with record frequency once every 0.1 s to calculate the real-time quantity of charge. Once the electrolysis was accomplished, the generalized products were esterified by adding CH_3_I (0.3 mL) and anhydrous K_2_CO_3_ (0.3 g) at 55 °C for 5 h. Subsequently, the solution was acidified with aqueous HCl (0.8 mol/L) and then extracted by diethyl ether. The organic layers were combined and then dried over anhydrous MgSO_4_. Then, the target product methyl benzoate (MB) was obtained after filtration and rotary evaporation. Finally, the identification of the target product MB was performed using GC–MS and comparing GC retention time for the target carboxylation product with that of a commercially available MB with a purity of 99%. The FE of the aim product MB was obtained through GC with the calibration curves (see Supplementary Figure S1 online). In the GC detection, the internal standard method was employed to calculate the quantification of MB, and decane was used as the internal standard.

## 4. Conclusions

In conclusion, we demonstrate a solar energy conversion device that produces high-value-added carboxylic acid ester MB from electrocarboxylation of BB with CO_2_ using photovoltaic technology under actual loads. Using two series-connected dye-sensitized photovoltaics and high-performance catalyst Ag electrodes, we achieved a 61.1% FE for MB. Stability experiments show that the FE is still close to the initial value after about 4 h. This study provides perspectives for obtaining high-value chemicals via solar-driven CO_2_ artificial photosynthesis and provides a new path for the conversion of CO_2_. However, despite the broad application prospects of this solar-driven artificial photosynthesis for CO_2_ electrocarboxylation, the cost of conductive glass is still relatively high. In addition, there is a risk of liquid leakage during the long-term operation of liquid DSCs, and the development of solid-state DSCs is also a trend in mass-scale applications.

## Data Availability

The data that support the findings of this study are available from the corresponding author upon reasonable request.

## Supplementary Materials

The following supporting information can be downloaded at: https://www.mdpi.com/article/10.3390/ijms251910608/s1.
