# Supplementary material for: Efficient Photosynthesis of Value-Added Chemicals by Electrocarboxylation of Bromobenzene with CO2 Using a Solar Energy Conversion Device
Source: Int J Mol Sci. 2024 Oct 1;25(19):10608. doi: 10.3390/ijms251910608 (PMC11476564; doi:10.3390/ijms251910608)
Supplement: Supplementary file 1 [file ijms-25-10608-s001.zip › ijms-3232745-supplementary.pdf]

Supplementary Materials

Efficient Photosynthesis of Value-Added Chemicals by Electrocarboxylation of Bromobenzene with CO_2 Using a Solar Energy Conversion Device

Yingtian Zhang, Cui Gao, Huaiyan Ren, Peipei Luo, Qi Wan, Huawei Zhou *, Baoli Chen * and Xianxi Zhang

Shandong Provincial Key Laboratory/Collaborative Innovation Center of Chemical Energy Storage, School of Chemistry and Chemical Engineering, Liaocheng University, Liaocheng 252059, China; lcuytz@126.com (Y.Z.); gaocuigc@163.com (C.G.); rhy17862548687@163.com (H.R.); luopeipeigood@163.com (P.L.); w15689397767@163.com (Q.W.); xxzhang3@126.com (X.Z.)

* Correspondence: zhouhuawei@lcu.edu.cn (H.Z.); chenbaoli@lcu.edu.cn (B.C.)

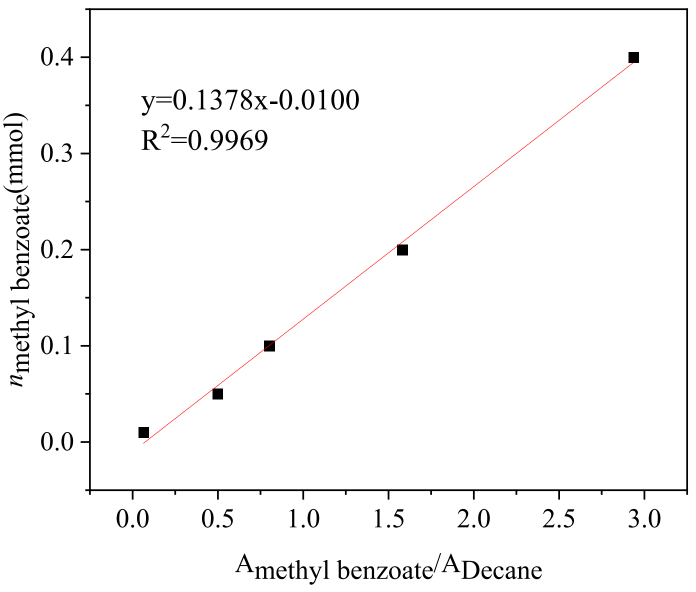

Figure S1. Standard curve for methyl benzoate quantitative analysis.

The standard curve of the carboxylation product methyl benzoate is shown in Figure S1. The mathematical expression of the standard curve is $y = 0.1378x - 0.0100$ ($R^2 = 0.9969$) and the linearity is good. Among them, y is the number of moles of methyl benzoate and x is the ratio of the peak area of methyl benzoate ($A_{methyl\ benzoate}$) to decane (A_{decane}) ($A_{methyl\ benzoate}/A_{decane}$). According to the standard curve equation, the FE of the target product methyl benzoate can be calculated.
